# Therapeutic strategies utilizing SDF-1α in ischaemic cardiomyopathy

**DOI:** 10.1093/cvr/cvx203

**Published:** 2017-10-13

**Authors:** Oliver J Ziff, Daniel I Bromage, Derek M Yellon, Sean M Davidson

**Affiliations:** The Hatter Cardiovascular Institute, Institute of Cardiovascular Science, University College London, 67 Chenies Mews, London WC1E 6HX, UK

**Keywords:** Stromal cell derived factor 1α, SDF-1α, CXCR4, Heart failure, Ischaemic cardiomyopathy

## Abstract

Heart failure is rapidly increasing in prevalence and will redraw the global landscape for cardiovascular health. Alleviating and repairing cardiac injury associated with myocardial infarction (MI) is key to improving this burden. Homing signals mobilize and recruit stem cells to the ischaemic myocardium where they exert beneficial paracrine effects. The chemoattractant cytokine SDF-1α and its associated receptor CXCR4 are upregulated after MI and appear to be important in this context. Activation of CXCR4 promotes both cardiomyocyte survival and stem cell migration towards the infarcted myocardium. These effects have beneficial effects on infarct size, and left ventricular remodelling and function. However, the timing of endogenous SDF-1α release and CXCR4 upregulation may not be optimal. Furthermore, current ELISA-based assays cannot distinguish between active SDF-1α, and SDF-1α inactivated by dipeptidyl peptidase 4 (DPP4). Current therapeutic approaches aim to recruit the SDF-1α-CXCR4 pathway or prolong SDF-1α life-time by preventing its cleavage by DPP4. This review assesses the evidence supporting these approaches and proposes SDF-1α as an important confounder in recent studies of DPP4 inhibitors.

## 1. Introduction

Modern therapeutic advances in treating ischaemic heart disease, including reperfusion strategies and secondary prevention, have improved patients’ survival. For example, primary percutaneous coronary intervention (PPCI) for ST-segment elevation myocardial infarction (STEMI) has led to a reduction in 30-day mortality from 13.7% in 1995 to 4.4% in 2010.^1^ This, combined with an aging population, has led to an increasing prevalence of ischaemic heart failure (HF), with current estimates suggesting that 26 million people live with HF worldwide and that myocardial infarction (MI) is a contributory factor in 29% of HF hospitalizations.^2^ Ischaemic cardiomyopathy describes significantly impaired left ventricular function resulting from coronary artery disease causing myocardial injury and ventricular remodelling. In contrast to the improvements in the treatment of many cardiovascular diseases (CVDs), survival rates of HF patients remain unacceptably poor with 1-year mortality following HF hospitalization after MI being 45.5%.^3^ Consequently, novel strategies to mitigate this burden of HF are paramount.

Stem cells are involved in the natural response to ischaemic tissue injury and have become a promising target of clinical research over the last decade, with the aim being to repair and replace damaged myocardium.^4^ Preclinical studies of various adult stem cells, including bone marrow (BM)-derived stem cells, endothelial progenitor cells (EPCs), and resident cardiac stem cells, have demonstrated beneficial effects on cardiac function and angiogenesis following MI, although clinical trial results have been mixed.^4^ It has been proposed that paracrine factors may mediate the favourable effects of stem cell engraftment. However, the duration of expression of these factors at the time of myocardial injury may be short.^5^ To improve stem cell mobilization and retention, and facilitate paracrine signalling, stem-cell homing signals from ischaemic cells are of considerable interest. Although many chemotactic factors are implicated, the chemokine stromal cell-derived factor 1α (SDF-1α/CXCL12) and its corresponding receptor CXCR4 have been identified as key regulators.^6^ SDF-1 is an 8 kDa CXC chemokine that comprises six alternatively spliced isoforms, of which SDF-1α is the principally expressed subtype. It is upregulated by hypoxia in a hypoxia-inducible factor 1 (HIF-1α)-dependent manner, and facilitates chemotaxis, stem-cell recruitment and cardiomyocyte survival via its G-protein coupled receptor, CXCR4^7^ SDF-1α and CXCR4 are up-regulated in the heart in both experimental and clinical studies of MI.^8^ In addition to mobilization and migration of stem cells, SDF-1α is also thought to confer direct protection against ischaemia-reperfusion (IR) injury via the same signalling pathways implicated in ischaemic conditioning.^7^ SDF-1α, therefore, exhibits pleiotropic effects on ischaemic myocardium: gradient-guided homing of stem cells towards sites of myocardial injury and direct protection via intracellular pro-survival signal transduction pathways.

Three approaches have been taken to optimize recruitment the SDF-1-CXR4 axis in the setting of ischaemic heart disease: (a) supplying artificial SDF-1α to match peak CXCR4 expression; (b) augmenting CXCR4 expression to meet the period of maximal SDF-1α release; and (c) minimizing SDF-1α degradation by dipeptidyl peptidase 4 (DPP4) and other proteases. Here, we review the role of SDF-1α in myocardial injury and examine the evidence that optimization of the SDF-1α-CXCR4 axis using these approaches may alleviate myocardial ischaemic injury.

## 2. SDF-1α-CXCR4 signalling

### 2.1 Normal signalling

Under hypoxic conditions HIF-1α upregulates both SDF-1α and CXCR4.^9^ In the hypoxic BM environment, BM stem-cells constitutively express CXCR4, which anchors them to the BM by SDF-1α expressed by stromal cells. SDF-1α degradation within the BM microenvironment causes mobilization of stem cells into the peripheral blood. Simultaneously, at the site of injury, a local rise in SDF-1α level recruits the mobilized cells from the circulation to the inflamed tissue. The CXCR4 antagonist, AMD3100, can also break this physical anchor in the BM, thereby enabling rapid mobilization of progenitor cells.^10^

In multiple experimental models of MI, SDF-1α is rapidly up-regulated and persists for 7 days in the infarct and peri-infarct zones (*Table 1*), thereby acting as a gradient-guided homing beacon to facilitate recruitment and adhesion of progenitor cells to the infarct border zone.^11^

**Table 1:** Preclinical studies examining the timing of SDF-1α and CXCR4 after ischaemia

Author	Model	Assayed	Method	Change in expression at different timepoints
Assaying SDF-1 in the Mouse				
Abbott, 2004^19^	Mo CAL	SDF-1 protein	ELISA. IHC localized expression to cardiomyocytes and blood vessels	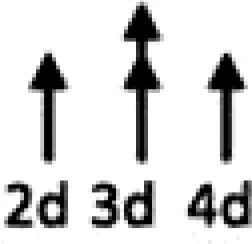
Abbott, 2004^19^	Mo CAL	SDF-1 mRNA	qRT-PCR	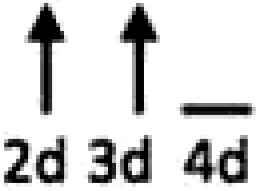
Kucia, 2004^20^	Mo IR	SDF-1 mRNA	qRT-PCR. IHC localized to cardiomyocytes and blood vessels	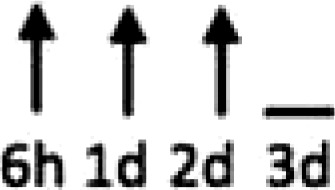
Assaying SDF-1 in the Rat				
Pillarisetti, 2001^9^	Rat CAL	SDF-1 mRNA	RT-PCT	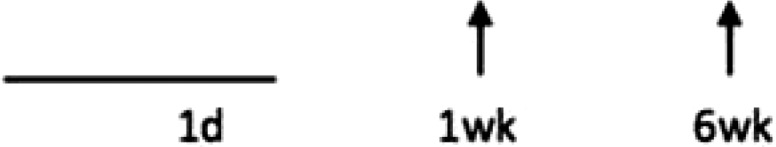
Askari, 2003^6^	Rat CAL	SDF-1 mRNA	RT-PCR	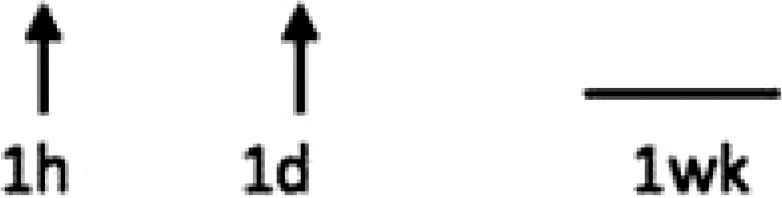
Czarnowska, 2007^21^	Rat CAL	SDF-1 protein	IHC	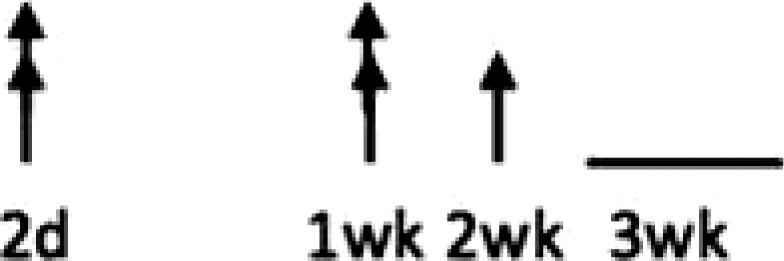
Segers, 2007^22^	Rat CAL	SDF-1 protein	ELISA	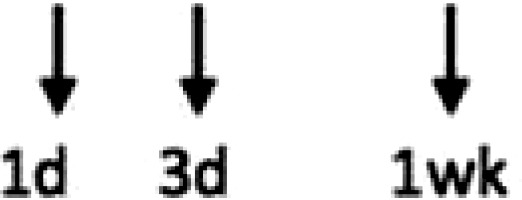
Misra, 2008^23^	Rat IR	SDF-1 protein	IHC. Localized to endothelium and infiltrating cells.	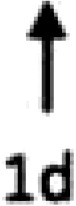
Assaying CXCR4 in the Rat				
Czarnowska, 2007^21^	Rat CAL	CXCR4 protein	IHC	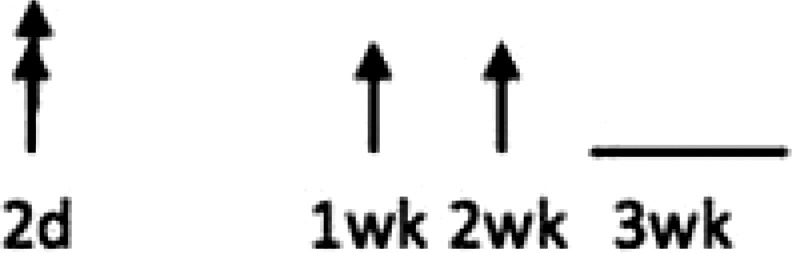
Misra, 2008^23^	Rat IR	CXCR4 protein	Radiotracer. IHC localized expression to cardiomyocytes	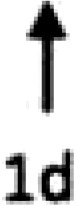
Zhang, 2007^24^	Rat IR	CXCR4 protein	IHC localized expression to cardiomyocytes	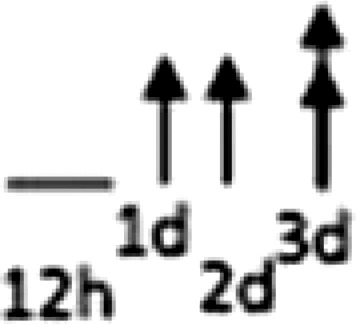

CAL, coronary artery ligation; IHC, immunohistochemistry; IR, ischaemia reperfusion; Mo, mouse; qRT-PCT, quantitative real time polymerize chain reaction.
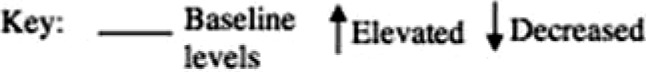

CXCR4 is expressed on stem-cells, peripheral blood leucocytes, endothelial cells and smooth muscle cells and cardiomyocytes.^12^^,^^13^ The binding of SDF-1α stimulates a G_i_ protein-pathway towards PLC-β and PI3K activation,^7^ as well as JAK/STAT, MAPK p42/44 extracellular signal-related (Erk1/2) and NF-ĸB pathways.^14^ CXCR4 signalling stimulates pathways that are important in cellular; (i) survival; (ii) proliferation and growth; (iii) chemotaxis; (iv) signalling and migration; and (v) adhesion and regulation of cytoskeletal apparatus.^14^ The effects are cell-type dependent, but are crucial in regulation of haematopoiesis, stem-cell homing, angiogenesis and cardiac repair.^6^^,^^12^^,^^13^ SDF-1α also binds a second GPCR called CXCR7, which was originally thought function as a non-signalling decoy co-receptor, but is now known to signal in its own right, primarily via β-arrestin and MAPK pathways.^15^ The physical and hormonal interaction of CXCR4 and CXCR7 and their impact these non-classical pathways makes the signalling role of SDF-1α on myocardial repair even more complex.^16^

### 2.2 Signalling in myocardial injury: a desynchronized orchestra

**Figure 1:**
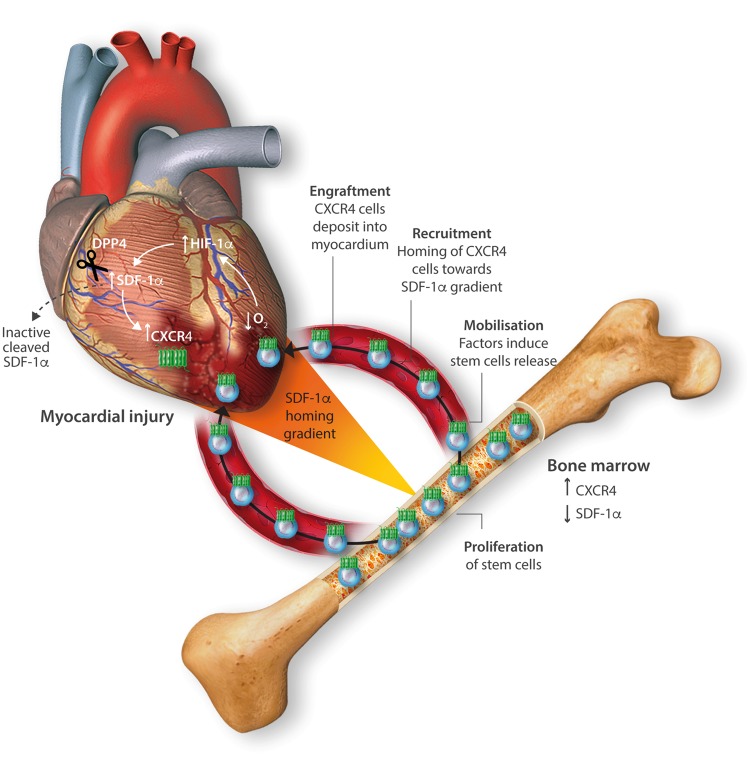
Mechanistic role of SDF-1α in response to myocardial injury. SDF-1α is increased in response to hypoxia via HIF-1, but is rapidly cleaved and inactivated by DPP4. Stem cells expressing CXCR4 are mobilized from bone marrow when SDF-1α levels decrease locally, and are recruited to areas of myocardium expressing SDF-1α.

Myocardial ischaemia results in elevated expression of both SDF-1α and CXCR4 in the myocardium, indicating that they might have a central role in the response to ischaemic injury. Additionally, platelet surface expression of SDF-1α, CXCR7 but not CXCR4 is significantly enhanced during ischaemia compared to stable coronary artery disease.^17^ CXCR4/SDF-1α signalling is required for progenitor cells to be recruited and increase angiogenesis and blood flow (*Figure 1*).^18^ Evidence from mouse models indicates that SDF-1α increases 6 h after ischaemic injury but only lasts for 3–4 days (*Table 1*).^19^^,^^20^ Data from rat models are conflicting with no clear reproducible time course of SDF-1α upregulation.^6^^,^^9^^,^^21–23^ However, there appears to be a delay in the CXCR4 time-course, which takes at least 1 day to increase and remains elevated for up to 2 weeks.^21^^,^^23^^,^^24^ Consequently, it has been postulated that CXCR4 upregulation has limited overlap with the SDF-1α surge.^25^ However, whether the same temporal mis-match applies to humans has yet to be established.

## 3. Retuning the SDF-1α-CXCR4 axis

### 3.1 Preclinical studies

#### 3.1.1 Artificially increasing SDF- 1α levels

Several approaches have been proposed to ‘retune’ the relationship between SDF-1α and CXCR4 after MI. Cardiac SDF-1α levels have been augmented using several strategies (*Table 2*). Direct intracardiac injection of SDF-1α protein in mice reduced infarct size, increased angiogenesis and improved cardiac function 4 weeks post-infarction,^11^^,^^26–28^ likely due to a combination of direct cardioprotection as well as stem cell recruitment. Timing of administration is likely crucial. In a pig model, SDF-1α injected into the peri-infarct zone 2 weeks post-MI did not improve infarct size or myocardial perfusion and actually impaired LV function.^29^ One approach to prolonging SDF-1α activity has been to bioengineer SDF-1α resistant to proteolytic cleavage. This improved stem-cell homing and myocardial retention, and also improved capillary density, blood flow and LVEF several weeks later.^22^

**Table 2:** Impact of artificially augmenting SDF-1 levels on infarcted myocardium

Author	Species	SDF-1 Dose and timing	Result	Mechanism
SDF-1 cardiac injection				
Koch, 2006^29^	Pig	2 weeks post MI, 18 x 5ig trans-endocardial injections into peri-infarct myocardium	Increased vessel density. Reduced cardiac function.	SDF-1 delivery associated with loss of collagen in peri-infarct area
Sasaki, 2007^26^	Mice	*CAL followed immediately by myocardial injection of 1ug SDF-1*	Improved function improved at 4 weeks. Smaller infarct size.	BM derived stem cells accumulated in SDF-1 myocardial injection site
Saxena, 2008^27^	Mice	2 x 300 ng SDF-1 intracoronary injection when ligated	Improved cardiac function after CAL at days 1–28	Akt activation in cardiac endothelial cells and cardiomyocytes
Tang, 2009^30^	Rat	CAL with immediate myocardial injection of 0.5 x 10^10^ pfu/mL Adenovirus-SDF-1.	Increased cardiac function at 4 weeks	Increased ckit^+^ stem cells recruited to infarcted area.
SDF-1 infusion				
Hu, 2007^11^	Mice	175 ug/kg perfusion into LV cavity then 10 min washout before IR	Reduced infarct size	Activated Aktp and Erkp
				Blocked by AMD3100
Huang, 2011^74^	Isolated Mouse heart	15-25ng/mL SDF-1 perfusion 5 min before ischaemia	Improved contractile function after IR	STAT3 increased but not PI3K or ERK1/2
Jang, 2012 ^75^	Isolated	25 nM pSDF perfusion at reperfusion	Reduced infarct size	Increased ERK1/2p no Aktp
	Rat heart			
Ziegler, 2012^76^	Mice	10 mg/kg SDF-1 intravenous infusion at d0 and d2 of CAL	Increased capillary density, reduced infarct size, preserved function	Enhanced recruitment of bone marrow stem cells
Stem cells over-expressing SDF-1				
Askari, 2003^6^	Rat	8 weeks post CAL cardiac fibroblasts with SDF-1 expression injected into myocardium	Increased vascular density	Increased haematopoietic stem cell recruitment to infarcted myocardium
			Improved LV function and strain.	
Deglurkar, 2006^33^	Rat	Transplanted SDF-1 expressing skeletal myoblasts 8 weeks post MI	Increased vascular density and cardiac function. Increased VT risk.	Not assessed
Elmadbouh, 2007^34^	Rat	Transfected SDF-1 into skeletal myoblasts	Increased vessel density. Improved LV function and remodelling.	Increased Aktp. Recruitment of stem cells into infarcted myocardium
Zhang, 2007^24^	Rat	MSC overexpressing SDF-1 infused 1 day post MI	Improved cardiac function at 5 weeks. Increased vessel density.	Preservation, not regeneration, of cardiac myocytes in the infarct zone.
Zhao, 2009^77^	Rat	MSC overexpressing SDF-1 injected into myocardial infarct region	Regeneration of cardiomyocytes. Increased vascular density.	Bone marrow progenitor cells recruited to infarct region.
Ischaemic preconditioning to increase SDF-1				
Hu, 2007^11^	Mouse myocytes	Ischaemic preconditioning increased SDF-1 three-fold.	Less injury after hypoxia/reoxygenation	Aktp and Erkp increased, JNKp and p38 decreased
Davidson, 2013^28^	Rat	Plasma SDF-1 increased after RIC (hindlimb 3x5 min cycles)	RIC decreased infarct size and improved cardiac muscle recovery	Improvements blocked by AMD3100.
Malik, 2015^78^	Human	25 ng/mL for 30 min prior to hypoxia/reoxygenation	Improved contractile function	Blocked by AMD3100

In a rat MI model, adenoviral delivery of SDF-1α post-infarction led to smaller infarct size, less fibrosis, more blood vessels, and improved LV parameters.^30^ Similarly, adenovirus-mediated cardiac expression of SDF-1α improved retention of BM-derived stem-cells (BMSC) delivered intra-coronary 48 h after MI.^19^ Human cardiac stem cells, engineered to overexpress SDF-1α and injected into infarcted mice, improved myocardial function and angiogenesis.^31^ Similar benefits have been observed after intracardiac injection of a variety of cells (fibroblasts, myoblasts, MSCs) overexpressing SDF-1α.^6^^,^^24^^,^^32–35^ However, CXCR4 may play a double-edged role, additionally contributing to inflammatory cell recruitment and remodelling processes after MI, since CXCR4^+/-^ mice have smaller infarct sizes than WT 4 weeks post MI.^36^

#### 3.1.2 Augmenting CXCR4 expression

CXCR4 expression has been augmented in stem cells with the aim of improving their cardiac recruitment. When MSCs overexpressing CXCR4 were delivered i.v. to rats 1–3 days post IR, recruitment to the infarct improved, as did neoangiogenesis, LV remodelling and function.^37^^,^^38^ Hypoxic culture increased CXCR4 expression in cardiosphere-derived, c-Kit^+^Lin^-^ stem-cells, and improved their cardiac recruitment after i.v. injection, reducing infarct size, increasing angiogenesis, and improving cardiac function.^39^

CXCR4 expression has also been increased in the myocardium. Adeno-associated viral vector (AAV9)-mediated over-expression of CXCR4 in the hearts of mice with trans-aortic constriction (TAC)-induced pressure overload preserved capillary density, prevented ventricular remodelling and maintained ventricular function.^40^ On the other hand, adenoviral delivery of myocardial CXCR4, prior to IR in rats was found to increase inflammatory cell infiltration and infarct area, as well as worsening cardiac function.^41^

These results suggest that re-synchronization of SDF-1α and CXCR4 expression after MI may be a valid approach, but that timing or method of delivery is crucial. Of note, these reports do not confirm that myocardial regeneration took place and do not distinguish between a direct effect on cardiomyocyte survival pathways and stem-cell-induced repair.^7^

#### 3.1.3 DPP4 inhibitors to extend SDF-1α half-life

A potential drawback with SDF-1α therapy is its relatively short half-life in plasma of 25.8 ± 4.6 min.^22^ Furthermore, this value represents total SDF-1α and does not distinguish between the active and cleaved, inactivated forms.^23^ The N-terminal lysine is rapidly cleaved by the protease DPP4, abolishing its bioactivity.^42^ Unfortunately, commercial antibodies recognize both the active and inactive forms and therefore report total levels of SDF-1α. A recently developed recombinant antibody recognizing only full length SDF-1α was identified in a phagemid library screen, and an ELISA based on this antibody should prove useful for quantifying active SDF-1α.^43^

The half-life of SDF-1α can be prolonged by inhibiting DPP4.^42^ DPP4 inhibitors (Sitagliptin, Vildagliptin, Alogliptin, and Saxagliptin) have become mainstay oral hypoglycaemic therapies in type 2 diabetes mellitus based on their capacity to prevent breakdown and prolong the activity of the incretin glucagon-like peptide 1 (GLP-1). However, less is known about exploiting these DPP4 inhibitors to increase the half-life of SDF-1α in ischaemic cardiomyopathy.

DPP4 inhibition was first shown to increase stem-cell homing to bone marrow.^44^ It also improved G-CSF-mediated stem-cell mobilization in a murine model of MI, improving cardiac remodelling, EF and survival.^45^ In a pacing-induced model of heart failure in pigs, Sitagliptin significantly improved stroke volume, heart rate, and the inotropic response to BNP.^46^ Similarly, Sitagliptin significantly improved cardiac function in a rat, LV-ablation model of HF.^47^

Interestingly, infarct size following IRI is reduced in DPP4 knockout mice or rats treated with Vildagliptin and Sitagliptin.^48–50^ Another target of DPP4, GLP1, may account for some of this protection,^49^ but the contribution of SDF-1α was not investigated in any of these studies. Furthermore, it is not clear whether DPP4 inhibition would compromise the longer-term, beneficial effects of the SDF-1α-CXCR4 axis with respect to ventricular remodelling.

### 3.2 Translating bench to bedside: SDF-1α in clinical studies

#### 3.2.1 Stem-cell based therapies

Since the encouraging early clinical trials of cell-based therapy for myocardial repair and regeneration, results have been conflicting and generally disappointing.^4^ One approach to improving the efficacy of stem-cell therapy is to increase the mobilization of endogenous stem-cells. Supporting this, in a study of 519 patients the number of circulating endothelial progenitor cells was correlated with improved LVEF and predicted the occurrence of CV events and mortality.^51^

Human BM harbours CXCR4^+^ progenitor cells, and these are mobilized into the peripheral circulation after MI, and migrate towards SDF-1.^20^^,^^52^ Interestingly, the infarct remodelling after intracoronary progenitor cell treatment in patients with acute myocardial infarction (TOPCARE-AMI) trial demonstrated that *in vitro* migration capacity of transplanted cells toward a gradient of SDF-1α was correlated with the reduction of infarct size assessed by MRI.^53^ Disappointingly, however, when CXCR4+ cells were selected from BM-derived progenitor cells and infused via the coronaries in a multicentre RCT of 200 patients with AMI (REGENT trial), they did not improve LVEF any more than non-selected cells.^54^

One explanation may be that injection of stem-cells in the days following an MI may partially miss the peak window of myocardial SDF-1α expression, leading to sub-optimal stem-cell homing. In addition, different cell isolation procedures may influence cellular CXCR4 expression.^55^

#### 3.2.2 Clinical studies of SDF-1α delivery

Attempts have been made to improve cardiac function by resynchronizing SDF-1α and CXCR4 expression following ischaemia in humans.

A naked DNA plasmid encoding SDF-1 (JVS-100) has been used to increase SDF-1 expression. This was found to be both safe and feasible, and encouragingly, in 17 patients with symptomatic ischaemic cardiomyopathy and LVEF <40% 6-min walk distance, NYHA class and quality of life was improved 1 year later.^56^ In a subsequent phase II double-blind RCT (STOP-HF), JVS-100 or vehicle was delivered via an endocardial catheter into the peri-infarct region in 93 patients with HF following MI. Included patients had LVEF ≤ 40% and were mostly NYHA class III (mean age 65 ± 9 years, 90% male) with baseline median NT-proBNP of 1000 ng/L and reduced exercise capacity. After 1 year, there was no difference in the primary endpoint of a composite score of 6-min walk distance and quality of life questionnaire at 4 months. There was no statistically significant difference in LV volumes or function at 1 year. However, in the pre-specified analysis, patients with LVEF <26% receiving 30 mg JVS-100 experienced an 11% increase in LVEF relative to placebo (*P* < 0.01).^57^ These results suggest that SDF-1 therapy may not only improve stem-cell homing many years following MI but may also induce reactivation of endogenous cardiac repair mechanisms. This study opens the door to regenerative gene therapies targeting endogenous stem cells and processes.

Several questions remain: (i) how long does SDF-1 expression remain active following delivery; (ii) do repeat treatments improve LV function and are these associated with an inflammatory type response; (iii) how does the time interval between the ischaemic insult and delivery impact on therapeutic response; and (iv) what is the optimal vector for delivery of SDF-1 to the myocardium? The FDA have approved STOP-HF 2, which will treat responsive patients identified in STOP-HF with 6-monthly repeat dosing.

#### 3.2.3 DPP4 inhibitors in HF

Interestingly, DPP4 may itself be implicit in the mechanism of heart failure. For example, circulating DPP4 activity correlates with cardiac dysfunction in human and experimental heart failure.^47^^,^^58^^,^^59^ In 14 patients with CAD and preserved LV function, inhibition of DPP4 with sitagliptin improved LVEF in response to stress testing, and mitigated post-ischaemic stunning.^60^ Accordingly, DPP4 inhibitors have been shown to improve CV outcomes.^47^^,^^58^^,^^61^ The target of DPP4 in HF may include BNP, GLP-1, and/or SDF-1. However, in type 2 diabetic patients, an increase in EPC mobilization after 4 weeks of Sitagliptin was associated with increased SDF-1α.^62^

Disappointingly, however, larger RCTs have failed to support a role for DPP4 inhibitors in CVD. All new oral hypoglycaemic agents for type 2 diabetes mellitus are required to undergo thorough CV safety evaluation. Consequently, three large multicentre clinical trials have recently demonstrated safety with regard to CV outcomes of DPP4 inhibitors in patients with type 2 diabetes at high risk for CV events (*Table 3*). However, SAVOR-TIMI 53 reported an increased risk of HF hospitalization in the Saxagliptin group compared to placebo (HR 1.27, 95% CI 1.07–1.51, *P* = 0.007).^63^ A subsequent sub-study found HF hospitalization to be highest in patients with elevated natriuretic peptides, previous HF or CKD.^64^ EXAMINE compared Alogliptin with placebo in 5380 patients with type 2 diabetes mellitus (T2DM) and recent acute coronary syndrome over median follow-up of 18 months and found no significant difference in the primary composite endpoint (CV death, non-fatal MI or non-fatal stroke) or in all-cause mortality or HF hospitalization.^65^ In a post-hoc analysis, there was no evidence of excess admissions for HF.^66^ The VIVIDD trial, which compared Vildagliptin with placebo in 254 patients with LV dysfunction (NYHA 1–3; LVEF <35%) and T2DM, reported no significant differences in HF hospitalization, LVEF or natriuretic peptide levels. However, the authors identified an increased LV end-diastolic volume and end-systolic volume with Vildagliptin compared to placebo.^67^ More recently, TECOS, which compared Sitagliptin to placebo in 14 671 patients with T2DM (HbA1c 6.5–8.0%) and CV disease, found no difference with respect to the composite primary outcome (CV death, nonfatal MI, nonfatal stroke, or hospitalization for unstable angina) or HF hospitalizations.^68^ In contrast to other DPP4 inhibitor trials, rates of HF hospitalization did not differ between groups, which may relate to baseline characteristic differences in patients enrolled, recording, and defining HF events or intrinsic pharmacological differences between DPP4 inhibitors.

**Table 3:** Major clinical trials investigating cardiovascular outcomes of DPP4 inhibitors

Study	Sample size	Population	Intervention vs. control	Follow- up (yrs)	Outcome (95% CI)
SAVOR-TIMI 53, 2014^64^	16, 492	T2DM, HbA1c 6.5-12.0%, >40 years with CVD OR men >55 or women >60 with dyslipidaemia, HTN or active smoking.	Saxagliptin 5mg o.d. (2.5mg if eGFR <50mL/min) vs. Placebo	2.1	Composite primary (CV death, nonfatal MI, nonfatal ischaemic stroke) HR 1.00 (0.89-1.12) All-cause death HR 1.11 (0.96-1.27) CV death HR 1.03 (0.87-1.22) MI HR 0.95 (0.80-1.12) Stroke HR 1.11 (0.88–1.39) Unstable angina hospitalization HR 1.19 (0.89-1.60) HF hospitalization HR 1.27 (1.07-1.51)
TECOS, 2015^79^	14, 671	T2DM receiving antidiabetic therapy, HbA1c 6.5-8.0% CVD, >50 years	Sitagliptin vs. Placebo	3.0	Composite primary (CV death, nonfatal MI, nonfatal ischaemic stroke) HR 0.98, 95% CI 0.89-1.08) All-cause death HR 1.01 (0.90-1.14) CV death HR 1.03 (0.89-1.19) MI HR 0.95 (0.81-1.11) Stroke HR 0.97 (0.79-1.19) HF hospitalization HR 1.00 (0.83-1.20)
EXAMINE, 2013^65^^,^^79^	5, 380	T2DM receiving antidiabetic therapy, HbA1c 6.5-11.0% (7.0-10.0% if on insulin), ACS within 15–90 days prior to randomization	Alogliptin 25mg (12mg if GFR <60; 6.25mg if GFR <30) vs. Placebo	1.5	Composite primary (CV death, nonfatal MI, nonfatal ischaemic stroke) HR 0.96, p = 0.32 All-cause death HR 0.88 (0.71-1.09) CV death HR 0.85 (0.66-1.10) Non fatal MI HR 1.08 (0.88-1.33) Non fatal stroke HR 0.91 (0.55-1.50) HF hospitalization HR 1.19 (0.90-1.58)
VIVIDD, 2013^67^	254	T2DM, HbA1c 6.5-10% (mean 7.8%), CHF NYHA 1-3, LVEF mean 30%,	Vildagliptin 50mg b.d. vs. Placebo	1	LVEF no difference LVEDV increased by 17.06mL vs. placebo (p < 0.05) LVESV increased by 9.44mL vs. placebo BNP -28% vs. -14% CV events no difference (35 vs. 31) CV mortality no difference (7 vs. 4 deaths) All-cause mortality no difference (11 vs. 4 deaths)
SITAGRAMI, 2016^69^	174	Revascularization after MI	Combined G-CSF and Sitagliptin vs. Placebo	1	LVEF -0.85% (-3.16-1.47%) RVEF 0.30% (-1.32-1.91%) MACE HR 0.79 (0.41-1.49)

The phase III clinical trial Safety and efficacy of SITAgliptin plus Granulocyte-colony-stimulating factor in patients suffering from Acute Myocardial Infarction (SITAGRAMI) randomized 174 patients to either G-CSF and Sitagliptin or placebo after PPCI for MI in a multi-centre, double-blind design. The primary endpoint of improved EF as assessed by magnetic resonance imaging at 6 months was not met, however, a non-significant trend towards reduced major adverse cardiac events was identified.^69^ This may be explained by the inclusion of only 21% of patients with LVEF below 50%, thereby obfuscating any potential benefit of this therapy.

**Figure 2:**
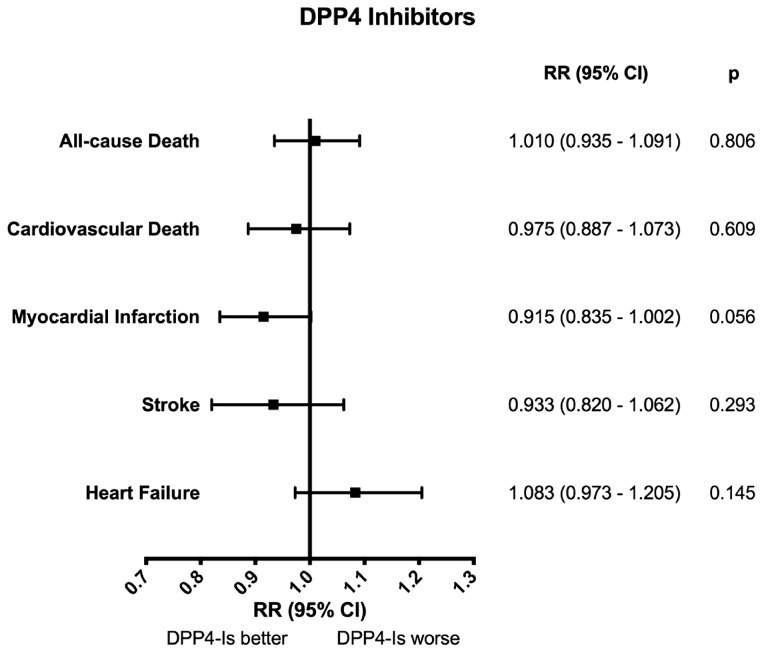
Meta-analysis of DPP4 inhibitors on cardiovascular events in patients with type 2 diabetes mellitus. Forrest plot showing the pooled clinical outcomes of 114 randomized trials comparing dipeptidyl peptidase 4 (DPP4) inhibitors with control (placebo or active drug). Reproduced from Ref. ^70^

Combined with mixed results from observational studies, the relationship between DPP4 inhibitors and HF is controversial. A recent comprehensive systematic review and meta-analysis of 114 randomized trials including 107 100 patients demonstrated that DPP4 inhibitors did not affect all-cause mortality (RR 1.01, 95% CI 0.94–1.09), CV mortality (RR 0.98, 95% CI 0.89–1.07), incident MI, stroke or HF.^70^ Although these trials achieved non-inferiority, they failed to demonstrate superiority with respect to clinical outcomes. Despite the finding in SAVOR-TIMI 53 that HF hospitalization increased with Saxagliptin, this meta-analysis suggested that DPP4 inhibitors, as a class, are safe in patients with high CV risk, and actually demonstrated a trend towards reduced MI (*Figure 2*). Importantly, in SAVOR-TIMI 53 there were key differences in baseline characteristics. Nonetheless, the US-FDA adverse event reporting system reported an association between Saxagliptin and HF.^71^ Additionally, a recent meta-analysis concluded that, despite an abundance of low-quality evidence, DPP4 inhibitors ‘may increase the risk of hospital admission for heart failure in those patients with existing CVD or multiple risk factors for vascular diseases, compared with no use’.^72^ However, the debate is far from over. Indeed, the most recent addition to the body of evidence is a population-based, retrospective cohort study of 255 691 South Korean patients with type 2 diabetes mellitus newly prescribed either DPP-4 inhibitors or sulfonylureas. This study found that DPP4 inhibitors significantly lowered future HF risk compared with sulfonylurea, and furthermore, that Sitagliptin and Linagliptin significantly lowered HF risk.^73^

Although the aforementioned studies were primarily based on the hypothesis that higher levels of GLP-1 would be beneficial, and none of them investigated SDF-1α, it is hypothesized that increased cleavage of SDF-1α after ischaemic injury is part of a reparative mechanism that, if interrupted by DPP4 inhibitors, may result in worse outcomes. The poor prognosis associated with high circulating DPP4 levels is likely to be related to reduced bioavailability of SDF-1α combined with direct adverse influences of DPP4 on fibrosis and inflammation. Thus, therapeutic use of DPP4 inhibitors to preserve SDF-1α and confer cardioprotection remains promising. However, future experimental and clinical research is required to decipher the appropriate time-course and clinical relevance in patients with HF.

## 4. Conclusion

The SDF-1α-CXCR4 axis plays a crucial role in homing stem-cells to ischaemic myocardium, resulting in the preservation and beneficial remodelling of myocardium. Since CXCR4 is expressed on BM stem-cells, SDF-1α offers a useful tool to remotely attract stem-cells to the site of injury. Based on a growing body of evidence, the complex dynamic signalling orchestra involved in the intricate network of cellular recruitment, migration, and engraftment to achieve myocardial repair is becoming clearer. This has exposed critical questions regarding optimal SDF-1α therapy including (i) timing; (ii) route of delivery; (iii) dosing regimen; (iv) duration of therapy; and (v) co-administration of DPP4 inhibitors to extend the half-life of SDF-1α.

Optimizing stem-cell homing and engraftment towards ischaemic myocardium by manipulating expression of migration signals is likely to be pivotal in the future of stem-cell therapy in HF. However, while DPP4 inhibitors may increase cardiac SDF-1α levels and enhance homing of circulating stem cells to the heart, they also reduce the number of BM stem cells mobilized and available for recruitment. Further experiments using tissue-specific knockouts are required to elucidate these mechanisms.

Marrying up the endogenous SDF-1α surge with CXCR4 upregulation appears crucial, but the precise timing in humans remains to be established. To this end, further work is required to establish the precise role of SDF-1α and subsequent recruitment of CXCR4 expressing stem-cells in clinical trials of DPP4 inhibitors. The recently development method for the direct measurement of the active (uncleaved) form of SDF-1α in blood should facilitate this task.^43^

**Conflict of interest:** none declared.

## Funding

This work was supported by a grant from the British Heart Foundation [Grant number PG/15/52/31598].
